# Kazakhstan tulips: comparative analysis of complete chloroplast genomes of four local and endangered species of the genus *Tulipa* L.

**DOI:** 10.3389/fpls.2024.1433253

**Published:** 2024-11-12

**Authors:** Dilnur Tussipkan, Vladislav Shevtsov, Malika Ramazanova, Aizhan Rakhimzhanova, Alexandr Shevtsov, Shuga Manabayeva

**Affiliations:** ^1^ Plant Genetic Engineering Laboratory, National Center for Biotechnology, Astana, Kazakhstan; ^2^ Plant Genomics and Bioinformatics Laboratory, National Center for Biotechnology, Astana, Kazakhstan; ^3^ Applied Genetics Laboratory, National Center for Biotechnology, Astana, Kazakhstan; ^4^ General Biology and Genomics Department, Faculty of Natural Sciences, L.N. Gumilyov Eurasian National University, Astana, Kazakhstan

**Keywords:** *Tulipa* L., chloroplast genome, comparative analysis, simple sequence repeat (SSR), codon usage patterns, phylogenetic analysis

## Abstract

Species of *Tulipa* are important ornamental plants used for horticultural purposes in various countries, across Asia, Europe, and North Africa. The present study is the first report on typical features of the complete chloroplast genome sequence of four local and endangered species including *T. alberti, T. kaufmanniana, T. greigii*, and *T. dubia* from Kazakhstan using Illumina sequencing technology. The comparative analyses revealed that the complete genomes of four species were highly conserved in terms of total genome size (152. 006 bp - 152. 382 bp), including a pair of inverted repeat regions (26. 330 bp - 26. 371 bp), separated by a large single copy region (82.169 bp - 82,378 bp) and a small copy region (17.172 bp -17.260 bp). Total GC content (36.58-36.62 %), gene number (131), and intron length (540 bp - 2620 bp) of 28 genes. The complete genomes of four species showed nucleotide diversity (π =0,003257). The total number of SSR loci was 159 in *T. alberti*, 158 in *T. kaufmanniana*, 174 in *T. greigii*, and 163 in *T. dubia*. The result indicated that ten CDS genes, namely *rpoC2, cemA, rbcL, rpl36, psbH, rps3, rpl22, ndhF, ycf1*, and *matK*, with effective polymorphic simple sequence repeats (SSRs), high sequence variability (SV) ranging from 2.581 to 6.102, and high nucleotide diversity (Pi) of these loci ranging from 0,004 to 0,010. For all intergenic regions longer than 150 bp, twenty one most variable regions were found with high sequence variability (SV) ranging from 4,848 to 11,862 and high nucleotide diversity (Pi) ranging from 0,01599 to 0,01839. Relative synonymous codon usage (RSCU) analysis was used to identify overrepresented and underrepresented codons for each amino acid. Based on the phylogenic analysis, the sequences clustered into four major groups, reflecting distinct evolutionary lineages corresponding to the subgenera *Eriostemons, Tulipa*, and *Orithyia*. Notably, *T. greigii* was distinctively grouped with species from *Orithyia* and *Eriostemons* rather than with other *Tulipa* species, suggesting a unique evolutionary history potentially shaped by geographical isolation or specific ecological pressures. The complete chloroplast genome of the four Tulipa species provides fundamental information for future research studies, even for designing the high number of available molecular markers.

## 1 Introduction

The *Liliaceae* family includes approximately 250 genera and 3500 species distributed worldwide (Ju et al., 2021). *Tulipa*. L. belongs to the subfamily *Lilioideae* and tribe *Lilieae*, and includes four subgenera *Clusianae*, *Tulipa*, *Eriostemones*, and *Orythia* (Christenhusz et al., 2013). The center of diversity of the genus is in the Pamir and Hindu Kush mountains and the steppes of Kazakhstan. Approximately 150 species of the genus *Tulipa* grow and develop well in the geographical regions of Asia, Europe, and North Africa (Marasek-Ciolakowska et al., 2018). *Tulipa* is a perennial plant that produces flowers in the spring during March to May. Tulips come in a wide range of colors, including red, pink, yellow, and white, with warm colors being the most common. Detailed accession-related passport information on 1257 *Tulipa* accessions is available on the online platform of the Genesys database and major *Tulipa* germplasm banks. The majority of accessions in the Genesys database are from Poland (636 accessions), followed by the Czech Republic (340 accessions), Ukraine (121 accessions), United Kingdom (72 accessions), Israel (64 accessions), United States (9 accessions), Russia (7 accessions), and Armenia (2 accessions) (https://www.genesys-pgr.org/). The Gardenia and Missouri Botanical Garden websites provide a comprehensive platform for exploring the botanical characteristics of various tulip species (https://www.gardenia.net, https://www.missouribotanicalgarden.org/).

Tulips are economically critical ornamental plants used for horticultural purposes in many countries (Marasek-Ciolakowska et al., 2018). In addition to their horticultural use, tulips have great value in the culinary world. Many varieties of edible flowers have been popular since ancient Greek and Roman times for enhancing the flavor of sweet and savory dishes (Rop et al., 2012). Tulip flowers are eaten to gain strength and as a classic dish for special occasions and have a wide range of medical and health benefits. For example, tulip flowers are the best remedy for coughs and colds, reduce the risk of cancer, used for sinus pain, hay fever and headaches; tulip flower extracts are an excellent poultice for insect bites, bee stings, burns, and rashes on the skin, as it gave quick relief with a soothing effect; tulip extracts have cosmetic uses in creams, hand lotions and in essential oils and perfumes. So, focusing on the nutritional and beneficial health benefits is essential. It is worth noting that not much research has been done on the medicinal use of *Tulipa* species.

The chemical composition of flowers from five *T. gesneriana* cultivars with different flower colors was analyzed for phenolic compounds (phenolic acids and flavonoids) and organic acids by Krzymińska et al. (Krzymińska et al., 2020). According to the results of this study, the total phenolic content in tulip petals is not affected by the place of cultivation, but the accumulation of organic acids in the petals is strongly correlated with the cultivars used, the duration of storage and the field or greenhouse conditions.

*Tulipa* species are believed to originate from Central Asia and is represented by 63 species in Central Asia (Vvedensky and Kovalevskaja, 1971). Scientists have confirmed that southern Kazakhstan and adjacent areas of Central Asia were the centers of origin of wild tulips (Rouhi et al., 2010). In the wonderful book «Tulips and other bulbous plants of Kazakhstan», published by Ivaschenko in 2005, a total of 34 species belonging to the three subgenera *Tulipa*, *Eriostemones*, and *Orythia* were described. These species are widely distributed throughout Kazakhstan (Ivaschenko, 2005). Eighteen species are listed in the Red Book and are protected by the state (https://astana.citypass.kz/en/2021/03/10/v-kazakhstane-35-vidov-dikih-tyulpanov/).This study discussed the typical characteristics of four local and endangered species of *Tulipa*, including *T. alberti*, *T. kaufmanniana*, and *T. greigii*. These species are listed in the Red Book and are protected at the state level.

*T. alberti* is native to Kazakhstan, Kyrgyzstan and Uzbekistan, and its distribution spans several regions of Kazakhstan, including the Karatau Mountains in the Syrdaria area, Chu Ili, the south of the Betpakdala desert, southwestern foothills of the Zhungar Alatau (in southern Kazakhstan). Its historical importance dates back to 1877, when Edward Regel described the flower of *T. alberti* based on samples collected by his son, Albert E. Regel, in the Karatau Mountains. The Karatau Mountains (are mainly located in the Zhambyl region of Kazakhstan, and the reference samples are now housed in the herbarium of the St. Petersburg Botany Research Institute. Morphologically, *T. alberti* is characterized by ovoid bulbs up to 3-4 cm in diameter, adorned with coriaceous, dark, fulvous, elongated scales. Its stems are robust, typically reaching 20 cm in height, while each bulb produces 3-4 linear, glaucous, wavy, broadly lanceolate, bluish-green leaves without spots (Zonneveld, 2009). The flower shows wide range of colors, from pure yellow and orange to mixed shades of crimson and incarnadine vermilion. Its fruits, up to 6 cm long and 2.5 cm wide, are produced from early April to the first decade of May, with fruiting occurring in May-June (Ivaschenko, 2005).

*T. kaufmanniana*, commonly known as the water lily tulip, is a species native to Central Asia, initially discovered in Turkestan region and described by Eduard August von Regel in the botanical journal “Gartenflora” in 1877 (POWO, 2024). This tulip species is well suited for rock gardens, beds, and borders. The bulbs of *T. kaufmanniana* vary in shape, from oblong to egg-shaped and turnip-like, up to 4 cm thick, covered with black-fulvous or golden-brown coriaceous scales. Their stems range from 10 to 50 cm in height, are often anthocyanin-colored, and typically bear two bluish grey leaves, occasionally three or four. Flower shapes vary greatly, from cup or wineglass to flat radial, with pointed or blunt tips of the perianth leaves. The colors are diverse, ranging from white, cream, gold, bright yellow, and orange to light red and burgundy. *T. kaufmanniana* blooms from late March to early July (Ivaschenko, 2005).

*T. greigii* was originally discovered in Turkestan region and widely distributed throughout Kazakhstan, from the northern deserts around Kyzylorda to the mountains and gentle foothills of Karatau. It is also found in the mountain ranges of West Tien-Shan, Kyrgyz, and Chu-lli, up to the Kordai Pass in the Ile Alatau Mountains, covering areas in Zhambyl, South Kazakhstan regions, and the eastern part of Kyzylorda region. It grows in loamy soils in valleys, foothills, and rocky slopes up to 2400 m altitude. The first sample of *T. greigii* was brought from the Karatau Mountains by Eduard August von Regel, who published its description in Gartenflora Vol. 22, on page 290 in 1873. The species was named after Samuel A. Greig (1827-1887), and the reference specimen was kept in the herbarium of the Botanical Research Institute of St. Petersburg. The bulb of *T. greigii* has ovoid bulb of up to 2-4 cm in diameter, 10-50 cm long fuzzy stem. The plant typically bears four leaves, although three or five leaves have also been observed, with leaf size decreasing towards the top. The lower leaf is oval-elliptic or broadly elliptic, while the upper leaf is spear-shaped. The leaves are bluish-grey with dark purple or violet spots of varying intensity. The flowers of *T. greigii* are usually single, quite large, and wineglass-shaped, reaching a height of 10-12 cm and a width of 10 cm. Their colors range from predominantly red with shades of orange, bright yellow, and light cream, with the undersides of red flowers being either black or yellow. The fruit can be up to 8 cm long and 25 cm wide. Propagation is primarily by seed, with cloning extremely rare (Ivaschenko, 2005). *T. greigii* flowers from early April to early June and bears fruit in June and July. The bulbs of this species can be eaten fresh or baked, and in Uzbek and Kazakh folk medicine, the petals are used to relieve headaches, while the seeds are used to treat boils.

*T. dubia* is endemic to the western Tian-Shan region and is widespread in Kazakhstan, Kyrgyzstan, and Uzbekistan. It grows mainly on the rubbly-melkosem slopes of the Tallas and Ugam ridges (Ivaschenko, 2005). It is distributed on dry, stony slopes and screes and typically inhabits the middle and upper mountain zones at altitudes from 1,500 to 3,300 meters above sea level. The *T. dubia* populations occur at higher altitudes compared to *T. kaufmanniana*. However, in the Aksay Valley, all three species discussed in this study grow at almost the exact altitudes but occupy different habitats. The plant is characterized by a short, perennial bulb that grows up to 20 cm high. Its broad green leaves are adorned with red stripes. The flower opens as a wide star with perigone segments that are 2-4 cm long, equal, yellow, red, or variegated yellow-red with a small indistinct yellow spot in red form or an orange spot in yellow form (Tojibaev et al., 2022).

It’s worth noting *T. alberti*, *T. kaufmanniana*, and *T. dubia* are listed as Critically Endangered or near-threatened on the IUCN Red List of Threatened Species, underscoring the importance of conservation efforts for these species. On the other hand, *T*. *greigii* was listed as Least Concern in the 2022 assessment, indicating a relatively stable population status for this species (https://www.iucnredlist.org/).

Chloroplast is an essential double membrane-bound organelle responsible for photosynthesis, primarily found in plant and algal cells. The complete chloroplast genome exists in both circular and linear configurations. It typically ranges in length from 120,000 and 170,000 base pairs (bp) and consists of two copies of inverted repeat regions (IRA and IRB) each spanning 20-28 kb, a large single copy (LSC) region of 80-90 kb, and a small single copy (SSC) region of 16-27 kb (Li and Zheng, 2018). The chloroplast genome comprises 120-130 genes, primarily involved in photosynthesis, transcription, and translation (Wicke et al., 2011; Turudić et al., 2023). Advances in high-throughput sequencing technologies have revolutionized the sequencing of chloroplast genomes due to their time-saving and cost-effective advantages. The complete chloroplast genome sequences of *Nicotiana tabacum* (tobacco) (Shinozaki et al., 1986) and *Marchantia polymorpha* (liverwort) (Ohyama et al., 1986) were first determined by Japanese scientists in 1986. Since then, numerous studies and reviews have discussed the structure and composition of chloroplast genomes from various land plant species (Wicke et al., 2011; Higgs, 2009; She et al., 2020; Lu et al., 2017; Daniell et al., 2016b; Ruang-Areerate et al., 2021; Dobrogojski et al., 2020; Tonti-Filippini et al., 2017). Chloroplast genomes have several favorable characteristics compared to nuclear genomes: their high abundance in the cell, small genome size, and haploid nature (Lu et al., 2017; Song et al., 2017). In addition, chloroplast genomes are predominantly maternally inherited (Park et al., 2021; Raspé, 2001; Villanueva-Corrales et al., 2021) although rare exceptions have been observed (Mccauley et al., 2007; Korpelainen, 2004). For example, Park et al. showed that the cucumber (*Cucumis sativus* var. *sativus*) chloroplast genome is maternally inherited in F1 hybrids, consistent with observations in other plant species (Park et al., 2021). However, investigations by McCauley et al. revealed rare non-maternal inheritance of chloroplast DNA in *Silene vulgaris* (Mccauley et al., 2007). Furthermore, chloroplast genomes exhibit highly conserved genome structure and gene order (Song et al., 2017; Lu et al., 2017; Chang et al., 2021; Ruang-Areerate et al., 2021). Despite their high conservation, chloroplast genomes exhibit significant microstructural variation at the boundaries of the four regions. As a result of these features, they serve as valuable sources for exploring neutral DNA markers for intraspecific and interspecific identification, evolutionary studies and phylogenetic relationships (Park et al., 2019a, Park et al., 2019b; Li et al., 2021a, Li et al., 2021b; Lu et al., 2017; Song et al., 2017). For example, Song et al. (2017); Lu et al. (2017), and Li et al. (2021c) studied the complete chloroplast genomes of three *Cardiocrinum*, three *Paris*, and five *Tulipa* species. They reported that while length, gene content, and gene order were nearly identical, these genomes exhibited nucleotide variability (Pi) in simple sequence repeats (SSRs, 1-10 bp), single nucleotide polymorphisms (SNPs), and long repeat sequences (greater than 30 bp) such as forward, palindromic, and complement repeats. Comprehensive studies have shown that microstructural variations are higher in non-coding regions than in coding regions (Li and Zheng, 2018). In addition to microstructural variation, major structural rearrangements occur during chloroplast genome evolution, including pseudogenization (Abdullah et al., 2021; Scobeyeva et al., 2021; Esfeld et al., 2018), gene deletions, and intron or exon losses (Frailey et al., 2018). Events such as the complete loss of one of the inverted repeats (Sabir et al., 2014), and large inversion of LSC and SSC regions as well as switching between these regions (Choi et al., 2021; Liang et al., 2022) have also been documented. These evolutionary processes contribute to the dynamic nature of chloroplast genomes and provide valuable insights into plant evolution and adaptation.

Whole chloroplast genome sequence analyses of species from different taxa are crucial for understanding chloroplast structures, gene organizations, diversity, genetic changes, recurrent adaptive evolution, and relationships among different groups of plant species (Ju et al., 2020a; Wicke et al., 2011; Ruang-Areerate et al., 2021; Fan et al., 2018; Cui et al., 2006). In addition, they can address challenging problems such the characterization of plastid-to-nucleus signaling mutants (Leister, 2003), plastome transformation (Olejniczak et al., 2016; Daniell et al., 2016b), developing chloroplast-derived vaccines against human diseases (Lössl and Waheed, 2011; Daniell et al., 2016a), understanding biogeographic history (Zhai et al., 2023), phylogeographic structure (Zhou et al., 2017) and obtaining information from plant fossils (Middleton et al., 2014).

The genus *Tulipa* contains about 150 species (Ju et al., 2021), however, based on the NCBI database, whole chloroplast genome sequences of several *Tulipa* species are available, including *T. altaica*, *T. thianschanica*, *T. iliensis*, *T. patens*, *T. sinkiangensis*, *T. schrenkii*, *T. gesneriana*, *T. buhseana*, *T. sylvestris*, *T. brachystemon*, *T. kolpakowskiana*, *T. fosteriana*, *T. zenaidae*, *T. alberti*, and *T. lemmersii* (Ju et al., 2021; Yuan et al., 2022; Ju et al., 2020b; Do et al., 2020; Zhou et al., 2019; Ju et al., 2020a; Li et al., 2021c). The present study is the first report on the complete chloroplast genome sequence of *T. alberti*, *T. kaufmanniana, T*. *greigii*, and *T. dubia* from Kazakhstan. The objectives of this study were (1) to determine and characterize the organization of the complete chloroplast genome sequence of *T*. *greigii*, *T*. *kaufmanniana*, *T. alberti* and *T. dubia* from Kazakhstan (2) to gain insight into the overall polymorphism and evolutionary dynamics of *Tulipa* chloroplast genomes; (3) to provide genes with effective SSRs and high nucleotide diversities for species identification in the genus *Tulipa*, and (4) to calibrate the phylogenetic position of *T. alberti*, *T. kaufmanniana*, *T*. *greigii* and *T. dubia* based on phylogenomic analysis by comparing with published complete chloroplast sequences of *Tulipa* species from the NCBI database. These results provide a more comprehensive understanding of the phylogeny of *Tulipa* and contribute primary genetic information for the phylogenetic relationship analysis of the genus and other relevant research.

## 2 Materials and methods

### 2.1 Materials

In this study, all plant materials of *T. alberti* (43^о^38’16’’ N, 68^о^37’46’’ E), *T. kaufmanniana* (42^о^20’51’’ N, 70^о^28’9’’ E), *T. greigii* (42^о^20’39’’ N, 70°25’29’’ E), and *T. dubia* (42^о^24’7’’ N, 70^о^35’43’’ E) were gathered at the collection sites in Karatau State Nature Reserve and Aksu-Zhabagly State Nature Reserve with the guidance of state reserve botanists in May of 2021 and 2022. Permission to collect samples of endangered species was obtained from the Forestry and Wildlife Committee of the Ministry of Ecology, Geology, and Natural Resources of the Republic of Kazakhstan. The detailed source information of species is described in **Table 1**.

**Table 1:** The detailed Source information and genome features of four species of *Tulipa* L. in this study.

Source information and genome features	*T. alberti*	*T. kaufmanniana*	*T. greigii*	*T. dubia*
**GenBank accession numbers**	OR458821	PP329299	PP335814	OR662047
**Coordinates**	43^0^38’16’’ N, 68^0^ 37’46’’ E	42^0^ 20’51’’ N, 70^0^ 28’9’’ E.	42^о^ 20’39’’ N, 70° 25’29’’ E	42^о^ 24’7’’ N, 70 ^о^ 35’43’’ E
**Altitude (m)**	710	2050	1830	1910
**Source**	Karatau Nature State Reserve	Aksu-Zhabagly Nature State Reserve	Aksu-Zhabagly Nature State Reserve	Aksu-Zhabagly Nature State Reserve
**Collection Date**	16.05.2022	14.05.2021	14.05.2021	19. 05.2022
**Genome size (bp)**	152,382	152,374	152,006	152,175
**SSC(bp)**	17,259 (1-17,259)	17,260 (1-17,260)	17,172 (1-17,172)	17,253 (1-17,253)
**IRA (bp)**	26,371 (17,260-43,631)	26,371 (17,261-43,632)	26,330 (17,173-43,503)	26,370 (17,254-43,624)
**LSC (bp)**	82,378 (43,632-126,010)	82,369 (43,633-126,002)	82,169 (43,504-125,673)	82,179 (43,625-125,804)
**IRB (bp)**	26,371 (126,011-152,382)	26,371(126,003-152,374)	26,330 (125, 674-152,006)	26,370 (125,805-152,175)
**Number of total genes**	131	131	131	131
**Protein-coding gene**	85	85	85	85
**tRNAs**	38	38	38	38
**rRNAs**	8	8	8	8
**A content (%)**	32.08	32.1	32.02	31.33
**T content (%)**	31.35	31.33	31.36	32.07
**C content (%)**	18.13	17.96	18.15	18.46
**G content (%)**	18.45	18.62	18.47	18.14
**Total GC content (%)**	36.58	36.58	36.62	36.60
**SSC GC content (%)**	29.85	29.85	30.01	29.88
**IR GC content (%)**	42.02	42.01	42.01	42.01
**LSC GC content (%)**	34.5	34.49	34.53	34.54

### 2.2 Plant material, DNA extraction and sequencing

Fresh leaves of *Tulipa* species were stored at -80°C until DNA was extracted. DNA was extracted following the protocol as described by Shi et al. (2012). The extracted DNA was checked for its intactness, homogeneity, and purity by 1% agarose gel electrophoresis and run at 120V for 30 min. The concentration and quantitative analysis were performed by NanoDrop™ 2000/2000c spectrophotometers (Thermo Fisher Scientific). To prepare the DNA libraries, Illumina^®^ DNA Prep, (M) Tagmentation (96 samples) (Illumina, 20018705) was used. Libraries were sequenced on the MiSeq sequencer using MiSeq Reagent Kit v3 (600-cycle) (MS-102-3003). All reagents and protocols were used according to the manufacturer’s instructions.

### 2.3 Chloroplast genome assembly and annotation

Raw data assembly was performed using Geneious Prime 2024.0.4 software (https://www.geneious.com). Genome annotation was performed using the MPI-MP CHLOROBOX GeSeq (Tillich et al., 2017) software tool based on the reference chloroplast genome of *T. altaica* (NC_044780.1), manually checked for errors and corrected. In addition, circular maps of the complete chloroplast genomes were illustrated by Chloe (Zhong, 2020) and were edited in Inkscape. Finally, the complete chloroplast genomes of four tulips were submitted to the NCBI Nucleotide database.

### 2.4 Analysis of chloroplast genome features

The alignment of four complete chloroplast genome sequences of *Tulipa* species was performed using BioEdit 7.7 (Alzohairy, 2011) and then subsequently used for further analyses. MEGA 11 (Tamura et al., 2021) software was used to calculate codon usage frequencies and relative synonymous codon usage (RSCU) values. RSCU values greater than 1.6 indicated overrepresented codons and RSCU values less than 0.6 indicated underrepresented codons (Noor et al., 2023). Next, boundary regions were visualized using IRscope (Amiryousefi et al., 2018) online software. All gene structures and varying relative positions near junctions were defined. Furthermore, Krait V1.5.1 (Du et al., 2017) was also used for the simple sequence repeat (SSR) identification and localization. The SSR sequences detected from the genome were divided into ten groups by length, 10 bp apart, namely 10-20, 21-30, 31-40, 41-50, 51-60, 61-70, 71-80, 81-90, >91 (Zhao et al., 2023). Genes with effective SSR-rich regions within the CDS and introns were selected and analyzed to identify divergent hotspots across *Tulipa* genomes). The value of nucleotide diversity (Pi) was calculated using DnaSP v5.10 (Librado and Rozas, 2009).

### 2.5 Characterization of sequence variability hotspot in CDS genes and intergenic regions

Sequences of each CDS and intergenic regions of more than 150 bp were blasted by the GenBank nucleotide BLAST of NCBI (https://blast.ncbi.nlm.nih.gov/Blast.cgi). Blast parameters were as follows: percent identity = 99-100, query coverage=99-100, and organism optional was Tulipa (taxid:13305). DnaSP v5.10.1 was used to sequence variability (SV) in each protein-coding gene (CDS) and intergenic regions. The number of mutations, indel events, conserved sites and nucleotide diversity (Pi) for each sequence were obtained by DnaSP (Librado and Rozas, 2009). SV was calculated according to the method of (Shaw et al., 2014; Smidt et al., 2020): SV = (number of nucleotide mutations + the number of indel events)/(the number of conserved sites + the number of nucleotide mutations + the number of indel events) × 100%.

### 2.6 Phylogenetic analysis based on SNPs

The whole chloroplast genome sequences of four *Tulipa* species in this study, together with previously published whole chloroplast genomes from NCBI were used to infer the phylogenetic relationships. SNPs were identified by comparing the mapped reads to the reference genome *T. altaica* (NC_044780) using BioNumerics. The tree was conducted using the Maximum Likelihood method and Kimura 2-parameter model in MEGA 11 with a bootstrap value of 500. The whole chloroplast genome of Smilax China (accession number: HM_536959) was used as an outgroup.

## 3 Results

### 3.1 Structure and content characteristics of the four *Tulipa* chloroplast genomes

**Figure 1:**
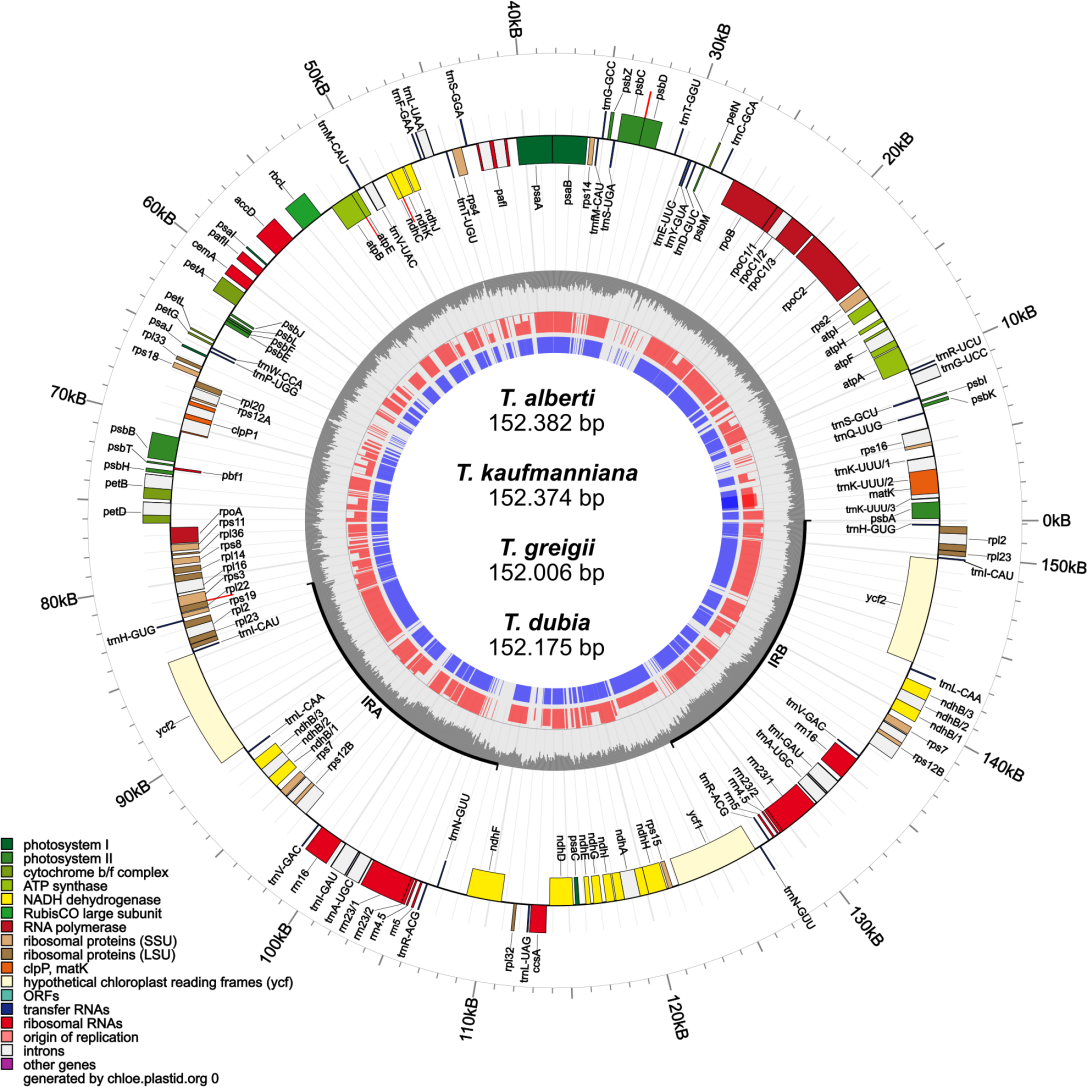
Chloroplast genome maps of four Tulipa species in this study.

The complete chloroplast genomes of *T. alberti* (152,382 bp), *T. kaufmanniana* (152,374bp), *T. greigii* (152,006 bp), and *T. dubia* (152,175 bp) were analyzed. The structure of these *Tulipa* chloroplast genomes consisted of a pair of IRA and IRB regions with lengths ranging from 26,330 to 26,371 bp, an SSC region with lengths ranging from 17,172 to 17,260 bp, and an LSC region with lengths ranging from 82,169 to 82,378 bp. The total GC content of *T. alberti* and *T. kaufmanniana* species were 36.58%, and that of *T. greigii* and *T. dubia* were 36.62% and 36.60%, respectively, in the whole genome. The GC contents of the SSC (29.85-30.01%) and LSC (34.49-34.54%) regions were almost identical among the species, while the IR regions showed higher GC contents, up to 42.02% (**Figure 1**; **Table 1**). A total of 151. 478 nucleotide pairs were analyzed for nucleotide pair frequency analysis, which revealed 99,64% identical pairs, 0,19% transitional pairs, and 0,17% transversional pairs in the four chloroplast genomes of *Tulipa* species (**Supplementary Figure S1**). Based on the results from Tajima's Neutrality Test, the nucleotide diversity ((π) was 0,003257. The annotated genome sequences of *T. alberti* (accession number OR458821.1), *T. kaufmanniana* (PP329299)*, T. greigii* (PP335814) and *T. dubia* (OR662047.1) are available in the NCBI database Further details on the genome characteristics are given in **Table 1**.

A total of 131 functional genes, including 85 protein-coding genes, were annotated in *T. Alberti* and *T. greigi*, while 130 genes, including 84 protein-coding genes, were annotated in *T. kaufmanniana* and *T. dubia* (**Table 1**). All these chloroplast genomes had 38 tRNA and 8 rRNA genes. In addition, all four genomes had 18 duplicated genes located in IR regions, including six coding genes (*ndhB*, *rpl2*, *rpl23*, *rps7*, *rps12B* and *ycf2)*, four RNA genes (*rrn4.5*, *rrn5*, *rrn16*, *rrn23)*, and eight tRNA genes (*trnA-UGC*, *trnH-GUG*, *trnI-CAU*, *trnI-GAU*, *trnL-CAA*, *trnN-GUU*, *trnR-ACG*, and *trnV-GAC)*. *ycf1* and *ycf2* were found in the genomes of *T. alberti*, *T. greigii*, *T. kaufmanniana*, and *T. dubia*, while *ycf15* and *ycf68* were pseudogenes in all four genomes (**Table 2**). The intron lengths (540 bp- 2620 bp) of 28 genes were well conserved across the four genomes (**Supplementary Table S1**). The *matK* gene was located in the intron of *trnK* gene in all four genomes.

**Table 2:** List of genes in the whole chloroplast genomes of four species of *Tulipa* L. in this study.

Category for genes	Group of genes	Gene names
**Self-replication**	Transfer RNAs	trnA-UGC*, *trnC-GCA*, *trnD-GUC*, *trnE-UUC*, *trnF-GAA*, *trnfM-CAU*, *trnG-GCC*, *trnG-UCC*, trnH-GUG*, trnI-CAU*, trnI-GAU*, *trnK-UUU*, trnL-CAA*, *trnL-UAA*, *trnL-UAG*, *trnM-CAU*, trnN-GUU*, *trnP-UGG*, *trnQ-UUG*, trnR-ACG*, *trnR-UCU*, *trnS-GCU*, *trnS-GGA*, *trnS-UGA*, *trnT-GGU*, *trnT-UGU*, trnV-GAC*, *trnV-UAC*, *trnW-CCA*, *trnY-GUA*
	Ribosomal RNAs	rrn4.5*, rrn5*, rrn16*, rrn23*
	RNA polymerase	*rpoA*, *rpoB*, *rpoC1*, *rpoC2*
	Small subunit of ribosomal Protein (SSU)	*rps2*, *rps3*, *rps4*, rps7*, *rps8*, *rps11*, *rps12A*, rps12B*, *rps14*, *rps15*, *rps16*, *rps18*, *rps19*
	Large subunit of ribosomal Protein (LSU)	rpl2*, *rpl14*, *rpl16*, *rpl20*, *rpl22*, rpl23*, *rpl32*, *rpl33*, *rpl36*
**Gene for photosynthesis**	Subunits of NADH-dehydrogenase	*ndhA*, ndhB*, *ndhC*, *ndhD*, *ndhE*, *ndhF*, *ndhG*, *ndhH*, *ndhI*, *ndhJ*, *ndhK*
	Subunits of photosystem I	*psaA*, *psaB*, *psaC*, *psaI*, *psaJ*
	Subunits of photosystem II	*psbA*, *psbB*, *psbC*, *psbD*, *psbE*, *psbF*, *psbH*, *psbI*, *psbJ*, *psbK*, *psbL*, *psbM*, *psbT*, *psbZ*
	photosystem I assembly factor	*pafI* (the former *ycf3*), *pafII* (the former *ycf4*)
	Subunits of cytochrome b/f complex	*petA*, *petB*, *petD*, *petG*, *petL*, *petN*
	Subunits of ATP synthase	*atpA*, *atpB*, *atpE*, *atpF*, *atpH*, *atpI*
	Large subunit of rubisco	*rbcL*
**Other genes**	Protease	*clpP1*
	Maturase	*matK*
	Subunit of acetyl-CoA-carboxylase	*accD*
	Envelope membrane protein	*cemA*
	C-type cytochrome synthesis gene	*ccsA*
**Genes of unknown function and pseudogenes**	Hypothetical chloroplast reading frames	*ycf1* and ycf2* appeared in all four genomes, while *ycf15* and *ycf68* were pseudogenes in all four genomes.

*Duplicated genes.

### 3.2 Comparison of LSC, IR, and SSC junction position among the four *Tulipa* species

**Figure 2:**
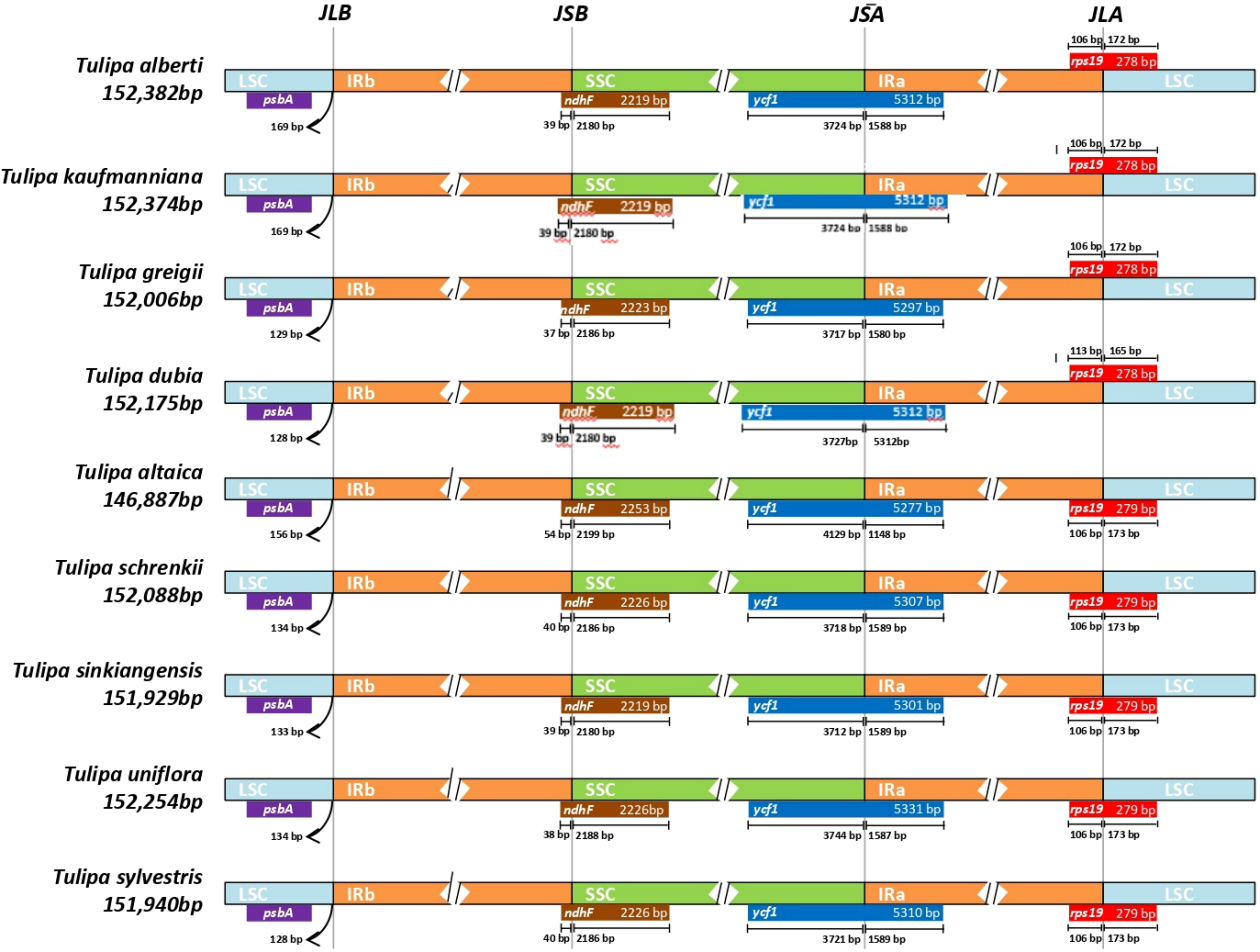
Comparison of SSC, IR, and LSC junction position among four *Tulipa* species. JSA, junction of SSC and IRA; JLA, junction of LSC and IRA; JLB, junction of LSC and IRB; JSB, junction of SSC and IRB.

Analysis of the IR/SC boundary areas for the four *Tulipa* chloroplast genomes was conducted by comparing them to closely related species within the common genus: *T. sinkiangensis* and *T. uniflora* from subgenus *Orithyia*, *T. sylvestris* from subgenus *Eriostemones*, *T. altaica* and *T. schrenkii* from subgenus *Tulipa* (**Figure 2**). In general, plastid genome regions’ lengths and gene number as well as order among 9 *Tulipa* species were conserved.

An identical haplotype was observed in within the SSC regions of *T. alberti*, *T. kaufmanniana*, *T. greigii*, and *T. dubia*, resulting in the maintenance of approximately equal amounts of base pairs, number of genes, and orientations. The *ycf1* gene was located in the boundary area of the IRA and SSC regions (JSA), while the *ndhF* gene was found in the boundary area of the SSC and IRB (JSB) regions of these species (**Figures 1**, **2**).

In the LSC regions of all four species, the same haplotype was identified, with approximately equal amounts of base pairs, number of genes, and orientations. The *rps19* gene was located in the boundary area of the LSC and IRA regions (JLA), ranging from 43,398 bp to 43,805 bp in the genome of these species. *psbA* gene was located in the boundary area of the IRB and LSC regions (JLB), ranging from 124,484 bp to 125,842 bp in the genome of these species (**Figures 1**, **2**).

### 3.3 Simple sequence repeat (SSR) pattern and variation analysis

**Figure 3:**
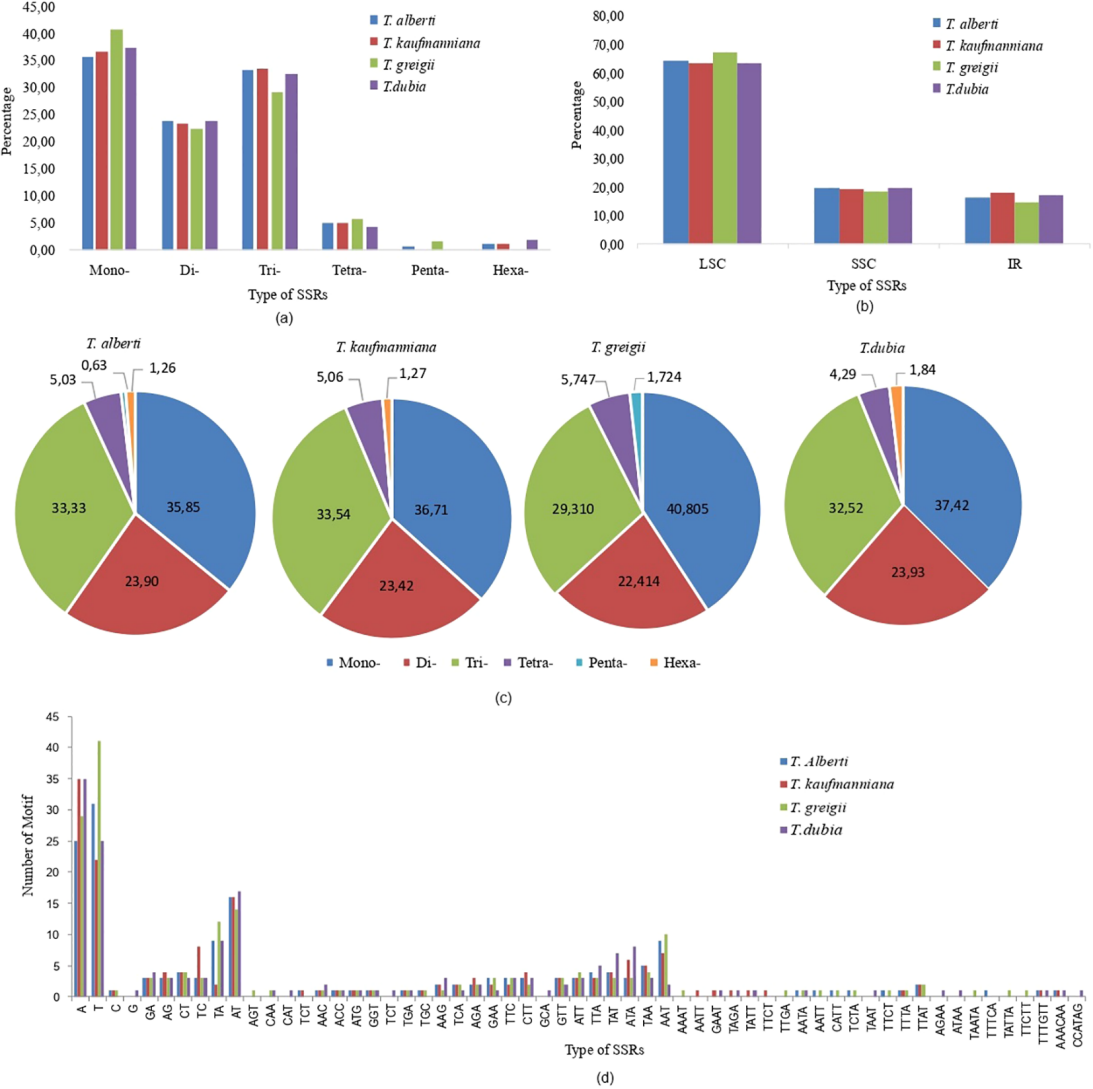
Type and number of identified SSR motifs (mono, di, tri, tetra, penta and hexa) in the whole chloroplast genome of four *Tulipa* species. **(A)** frequency of different SSR motif types in the whole chloroplast genome; **(B)** frequency of Identified SSR motifs in the SSC, IRA, LSC, and IRB regions; **(C)** percentage of mono, di, tri, tetra, and penta SSR motifs in each chloroplast genomes; **(D)** type and number of identified SSR motifs (mono, di, tri, tetra, penta, and compound).

The total number of SSR loci was 159 in *T. alberti*, 158 in *T. kaufmanniana*, 174 in *T. greigii*, and 163 in *T. dubia*. SSRs are repeated DNA sequences consisting of tandem repeats of 10-20 in length per unit distributed throughout the genomes of all four *Tulipa* species, followed by 21-30 bp and 31-40 bp in length. Through comparative genomic analysis, abundant SSRs were detected in the LSC region ranging from 63.19% to 67.2%, followed by the SSC region ranging from 18.39% to19.63% and the IR region ranging from 14.37% to 17.72%. There were 59 types and six categories of SSRs (mono-, di-, tri-, tetra-, penta-, and hexanucleotide repeats) in the chloroplast genomes of studied *Tulipa* species. The distribution of motif types consisted of 57-70 mono, 37-39 di, 51-53 tri, 7-10 tetra, 1-3 penta, and 2-3 hexa SSRs. The frequency of occurrence was 35.85-40.80% for mono, 22.41-23.93% for di, 29.31-33.54% for tri, 5.03-5.75% for tetra, 0.63-1.72% for penta, and 1.26-1.84% for hexa SSRs (**Figure 3**; **Supplementary Table S2**).

#### 3.3.1 Common types of SSRs in the four genomes of *Tulipa* species

Twenty types of SSRs, including two types of monomers, six types of dinucleotides, and eighteen trinucleotides, were found in all chloroplast genomes of *Tulipa* species. T and A nucleotides were reported as the most abundant motifs, according to 35.22%, 36.08%, 40.23%, and 36.81% of all SSR motifs in the chloroplast genomes of *T. alberti*, *T. kaufmanniana, T. greigii*, and *T. dubia*, respectively. The major types of dinucleotide SSRs were AT and TA, followed by TC, CT, GA, and AG. The most abundant trimotifs were AAT, TAA, TTA, TAT, TTC, GTT, GAA, CTT, ATT, ATA, TCA, AGA, AAG, TGA, GGT, ATG, ACC and AAC.

#### 3.3.2 Common types of SSRs in the three genomes of *Tulipa* species

Monomers of type C, trimotifs of type TGC, tetranucleotide repeats of type TTAT and TTTA were common for the genomes of *T. alberti*, *T. kaufmanniana*, and *T. greigii*.

#### 3.3.3 Common types of SSRs in the two genomes of *Tulipa* species

The TCT type of tri-motif was found in the genomes of *T. albert* and *T. kaufmanniana*, while the CAA of tri-motif was found in the genomes of *T. greigii* and *T. dubia*. The tetranucleotide repeats, including AATA, AATT, CATT, TCTA, and TTCT, were distributed in the genomes of *T. alberti* and *T. greigii*, and GAAT, TAGA, and TATT were distributed in *T. kaufmanniana*, and *T. dubia*. Only three types of hexanucleotide repeats were found, among which TTTGTT and AAACAA were distributed in the genomes of *T. alberti* and *T. kaufmanniana.* No hexanucleotide repeats were found in the genome of *T. greigii.*

#### 3.3.4 Unique SSRs only found in one genome of *Tulipa* species

The AGT type of tri-motif was found in *T. greigii.* G type of monomer, CAT, TCT, and GCA type of tri-motifs, AGAA, ATAA, and TAAT of tetranucleotide repeats and CCATAG type of hexanucleotide repeats were found in only *T. dubia.*

#### 3.3.5 SSRs in the intergenic, protein coding gene sequences (CDS), intronic, tRNA regions

The intergenic regions of the four genomes of *Tulipa* species had the highest SSR density, ranging from 84 to 102, followed by CDS (38-64), intron (16-22), and tRNA (1-3). *T. dubia* had the most intergenic and tRNA SSRs, *T. greigii* had the most CDS SSRs, and *T. alberti* had the most intronic SSRs (**Supplementary Figure S2**).

#### 3.3.6 Characterization of sequence variability hotspot in CDS genes and intergenic regions

**Figure 4:**
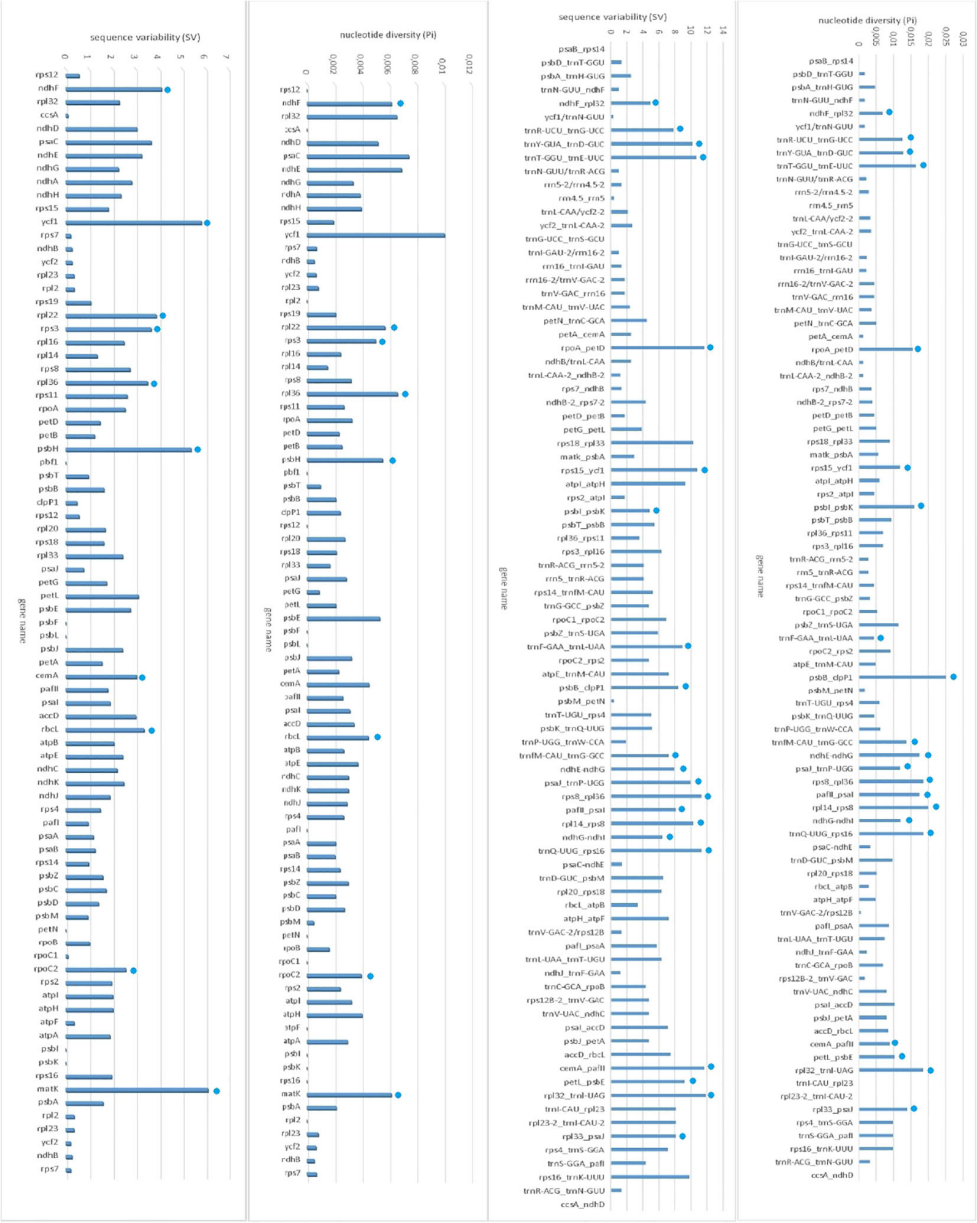
Sequence variability (SV) and nucleotide diversity (Pi) within each protein-coding sequences (CDS) and intergenic regions.

We found a total of 10 CDS genes that have effective polymorphic SSRs, with high sequence variability (SV) ranging from 2.581 to 6.102 and high nucleotide diversity (Pi) of these loci ranging from 0,004 to 0,010. They are *rpoC2, cemA*, *rbcL, rpl36, psbH, rps3, rpl22, ndhF*, *ycf1* and *matK* (**Supplementary Table S3**) (**Figure 4**).

For all intergenic regions longer than 150 bp, twenty one most variable regions were found with high sequence variability (SV) ranging from 4,848 to 11,862 and high nucleotide diversity (Pi) ranging from 0,01599 to 0,01839. They are *ndhF_rpl32*, trnR-*UCU_trnG-UCC*, *trnY-GUA_trnD-GUC*, *trnT-GGU_trnE-UUC*, *rpoA_petD, rps15_ycf1, psbI_psbK*, *trnF-GAA_trnL-UAA*, *psbB_clpP1*, *trnfM-CAU_trnG-GCC, ndhE-ndhG, psaJ_trnP-UGG, rps8_rpl36, pafII_psaI, rpl14_rps8, ndhG-ndhI, trnQ-UUG_rps16, cemA_pafII, petL_psbE, rpl32_trnl-UAG*, and *rpl33_psaJ* (**Figure 4**).

### 3.4 Codon usage patterns

**Figure 5:**
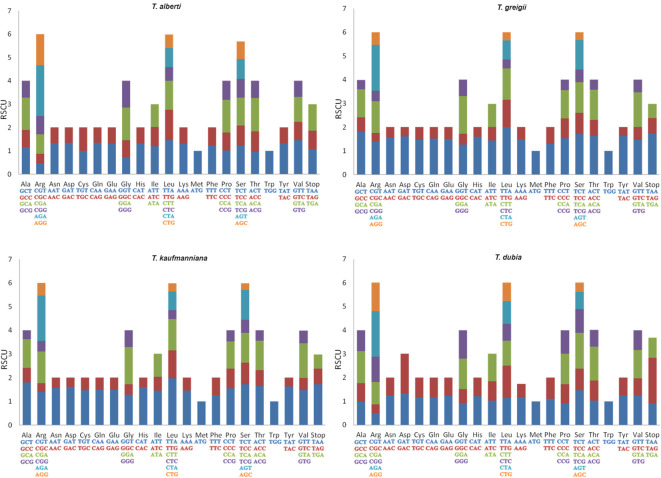
Codon content of 20 amino acids and stop codons in the CDS of four *Tulipa* species. The color of the histogram corresponds to the color of the codons.

The CDS of four *Tulipa* species were used for codon usage analysis. The total number of codons in the genomes of the analyzed species ranged from 22,457 (*T. dubia*) to 25,695 (*T. greigii*) (**Supplementary Figure S3**). The third most abundant codons were T (36.065%), followed by A (31.065%), G (17.02%), and C (15.85%) in the genomes *T. alberti*, *T. kaufmanianna* and *T. greigii*, whereas in the genome of *T. dubia*, A (29.68%) was the most abundant codons followed by T (26.65%), G (24.6%), and C (19%). Identified twenty-one high frequency (HF) codons (RSCU>1) were general for all Tulip species (**Figure 5**). Two HF codons (RSCU>1), GCT and CTT, were identified in the genomes of *T. alberti*, *T. kaufmanianna* and *T. greigii*. ACT (RSCU>1) was found in *T. kaufmanianna*, *T. greigii*, and *T. dubia*. GGG and AGG (RSCU>1) were identified in the genomes of *T. alberti* and *T. dubia*. Five HF codons (RSCU>1), including TTA, CGA, CGT, GGT, and AGT were found in *T. kaufmanianna* and *T. greigii*. Four unique codons (RSCU>1), namely GTT, TCG, TGT, and ATA, were found only in the genome of *T. dubia*. Seven codons (TTA, AGA, GCT, TCT, TAT, ACT, and GAT) were considered overrepresented codons (RSCU>1.6) in the genomes of *T. greigii* and *T. kaufmanianna.* In contrast, only one (AGA) overrepresented codon (RSCU>1.6) was found in the genomes of *T. alberti* and *T. dubia.* Twenty-four underrepresented (RSCU<0.6) codons were found in the genomes of *T. greigii* and *T. kaufmanianna*. They are AAC, AAG, ACG, AGC, AGG, ATC, CAC, CAG, CCG, CGC, CGG, CTC, CTG, GAC, GAG, GCG, GGC, GTC, GTG, TAC, TCG, and TGC. Five underrepresented (RSCU<0.6) codons, such as AGC, CGC, CGT, CTC and CTG were found in the genomes of *T. alberti* and *T. dubia*. According to the codon usage analysis, at amino acid levels, Leucine was the most abundant amino acid in the genomes of *Tulipa* species, with an average of 11.51%. Cysteine was the least abundant amino acid in the genomes of *T. greigii*, *T. kaufmanianna*, and *T. dubia*, with an average of 1.38%, and methionine was the least frequent amino acid in the genome of *T. alberti.* The results of the total RSCU values are shown in **Supplementary Table S4**.

### 3.5 Phylogenetic analysis

**Figure 6:**
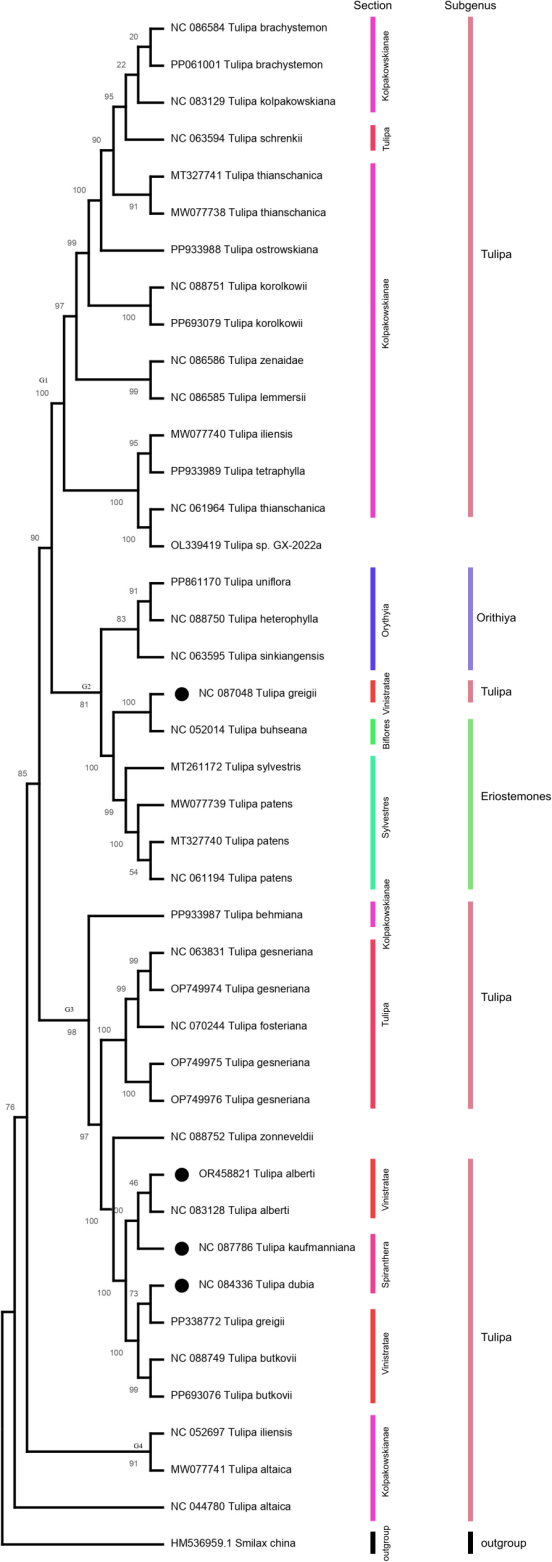
Phylogenetic relationship for *Tulipa* L. species. Inferred from Maximum likelihood model.

According to SNP-based on the phylogenic analysis, all 40 sequences across 25 species were clustered into four main groups (**Figure 6**). Group 1, group 3, and group 4 contained species of subgenus *Tulipa*, while group 2 contained species of subgenus *Orithyia* and *Eriostemons*.

Three *Tulipa* species including *T. alberti, T. kaufmanniana*, and *T. dubia* from Kazakhstan were formed one group with species from subgenus *Tulipa* and the sections *Tulipa* and *Vinistriatae*, however, *T.greigii* in formed a separate with species from subgenus *Orithyia* and *Eriostemons.*

## 4 Discussion

### 4.1 Structure and content characteristics of the four *Tulipa* chloroplast genomes

The results presented in this study provide a comprehensive analysis of the chloroplast genomes of four *Tulipa* species, namely *T. alberti*, *T. kaufmanniana*, *T. greigii*, and *T. dubia*. These genomes shared typical structural features, including a pair of IR, SSC, and LSC regions, with slight vitiations in the lengths ranging from 152,006 bp to 152,382 bp. Consistency in chloroplast genome sizes across all species in the *Liliacae* family has been observed previously in other studies (Li et al., 2021c; Lu et al., 2017; Do et al., 2020). All *Tulipa* species that we analyzed from NCBI database for phylogenetic relationship had whole genome sizes between 151,691 bp and 152,382 bp, except for *T. altaica* (146,887 bp, NC_044780) and *T. fosteriana* (153,308 bp, NC_070244). The GC content of the chloroplast genomes was consistent across species, with minor variations (36.58-36.62%) observed. Notably, the GC content was higher in the IR regions compared to the SSC and LSC regions. This phenomenon is consistent with findings in other plant species (Li et al., 2021d; Jiang et al., 2023; Li et al., 2022b) and is thought to be related to the IR regions’ functional significance and structural constraints. Nucleotide pair frequency analysis reveals the distribution of identical, transition, and transversion pairs, and the nucleotide diversity of the four chloroplast genomes showed differences, providing insight into the genetic diversity within these genomes.

In this study, based on the relative order and the orientation of the genes, identical haplotypes were identified in the SSC and LSC regions. There were no detected inversion events in the regions of all four species. However, inversion events in the SSC regions were first reported in the chloroplast genomes of *Gossypium barbadense* (Ibrahim et al., 2006), *Phoenix dactylifera* (Yang et al., 2010), *Artemisia frigida* (Liu et al., 2013), *Lasthenia burkei* (Walker et al., 2014), and various *Asteraceae* species (Zhang et al., 2014). Despite being overlooked in several studies, Walker et al. discussed the sources of inversion variation in the SSC region of chloroplast genomes in the American journal of botany in 2015 (Walker et al., 2015). Three structural haplotypes were reported in 61 land plant species from 19 orders of angiosperms, gymnosperms, and pteridophytes. Wild variation in the orientation of the SSC region was found in the *Malvaceae* family (Cheng et al., 2020). Using the relative order of genes, Liang et al. (2022) revealed SSC region switching in the chloroplast genome of wild rice (Liang et al., 2022).

Inversion events in the LSC regions have also been reported in many plant studies. In addition, the occurrence of flip-flop recombination on short inverted repeat sequences has been demonstrated, generating different isoforms of the transformed plastid genome that differ in the orientation of a 70 kb segment in the LSC region of tobacco (Rogalski et al., 2006). A structural inversion occurred in the LSC region of wild rice, resulting in the reverse orientation of 36 genes (Liang et al., 2022). The LSC region between the *accD* and *rps16* genes contains a 54 kb inversion, the most prominent difference in the chloroplast genome of *Oenothera elata* (Hupfer et al., 2000). Three inversions (Inversion I, rps4; Inversion II, trnH-GUG/rps16; and Inversion III, trnS-GCU/trnS-GGA) occurred in *Anemoclema*, *Anemone*, and *Pulsatilla* (Choi et al., 2021). A total of 35 gene inversions spanning 44.8 kb, including 21 protein-coding genes and 14 tRNAs from trnT-UGU to rps16 genes, were detected in the chloroplast genome of *Adonis mongolica* (Nyamgerel et al., 2023).

Ibrahim et al. (2006) suggested that the faster divergence of SSC and LSC regions than IR regions may be caused by the differences between their introns and intergenic regions. Liang et al. (2022) explained it by comparing it with the analogous to the mating-type (MAT) region of a yeast genome in the study of Haber (2012) and suggested that SSC switching, MAT switching, or even more transposon-like elements may share common mechanisms. All research studies attempted to explain this phenomenon, generally attributing it to the results of intramolecular recombination event (Ibrahim et al., 2006; Liu et al., 2013; Walker et al., 2015).

### 4.2 CDS Genes and intergenic regions could be used as novel chloroplast markers for species identification in the genus *Tulipa*

The results of previous studies have shown that comparative analyses of chloroplast genome sequences of species in a family help to identify sequence variability (SV) and nucleotide variability and subsequently to determine highly variable genes and intergenic regions as genetic markers used for genetic or phylogenetic studies (Hong et al., 2020; Li et al., 2021c; Cheng et al., 2020; Fan and Ma, 2022). In this study, the comparative analysis of four *Tulipa* species and species from NCBI showed that ten genes, and twenty-one intergenic regions with high nucleotide diversity (Pi) and sequence variability (SV) could be used as molecular markers for future phylogenetic analysis and species identification studies in the *Liliaceae* family.

These genes, including *rpoC2, rbcL, psbH, rpl22, ndhF*, *ycf1*, and *matK* have been proposed as high divergence and novel chloroplast markers for species identification, discrimination, and in *Tulipa* (Li et al., 2021c; Almerekova et al., 2024; Sutula et al., 2024), Poaceae (Moon et al., 2016), *Meconopsis* (Li et al., 2019), Orchidaceae (Huyen-Trang et al., 2017), Siraitia Merrill (Huyen-Trang et al., 2017), *Paris* (Song et al., 2017) species. There was no information about *cemA*, *rpl36* and *rps3* used as markers.

*ndhF_rpl32*, trnR-UCU_trnG-UCC, *rpoA_petD, rps15_ycf1, ndhG-ndhI, trnY-GUA_trnD-GUC, ndhE-ndhG*, *psaJ_trnP-UGG, trnT-GGU_trnE-UUC, trnQ-UUG_rps16, rpl14_rps8, cemA_pafII*, *rpl33-psaJ*, and *petL_psbE* were most variable loci in *Adenophora racemose, Iridaceae* species, *Broomcorn Millet*, *Glycyrrhiza eurycarpa*, *Camellia ‘Xiari Qixin*’, *Apiaceae* species, *Primula sinensis* (Primulaceae), *Hibiscus sinosyriacus*, *Atropa vs. Nicotiana, Lotus vs. Medicago*, and *Saccharum vs. Oryza, Medicago sativ* (Kim and Cheon, 2021; Xiao et al., 2024; Oyuntsetseg et al., 2024; Nie et al., 2018; Zhang et al., 2022; Elyor et al., 2023; Xu et al., 2023; Gou et al., 2020; Kwon et al., 2023; Shaw et al., 2007; Li et al., 2022a).

*psbI-psbK* (Baraket et al., 2011), *trnL (UAA)-trnF (GAA)* (Sen et al., 2020; Abdessamad et al., 2016; Baraket et al., 2008), *rps8_*rpl36 (Seberg and Petersen, 2009), *rpl14-rps8* (Ikinci et al., 2011), were used as robust markers for sequence variations, molecular evolution phylogenetic analysis in *Pisum sativum* L. *Palisota* (Commelinaceae), *Ficus carica* L*., Quercus suber* L., *Crocus* L., *Scorpiris*. To our knowledge, *trnfM-CAU_trnG-GCC* and *pafII_psaI* were found as newly explored regions.

### 4.3 Codon usage patterns

SCUB analysis is a powerful tool for investigating species specificity, evolutionary relationships, mRNA translation, and discovering novel genes, all of which are important for understanding gene function and molecular phylogeny (Song et al., 2022). This study analyzed the CDS of four *Tulipa* species to investigate codon usage patterns and amino acid frequencies. The total number of codons in the genomes of the four species ranged from 22,457 to 25,695, indicating slight differences in genome size among the species. Interestingly, the distribution of codons showed different patterns among the species. For example, in the genomes of *T. alberti*, *T. greigii*, and *T. kaufmanianna*, the most abundant third codon base was T, followed by A, G and C. In contrast, in the genome of *T. dubia*, it was A, followed by T, G, and C. This variation suggests potential differences in evolutionary pressures and genome composition among the species. In *T. greigii* and *T. kaufmanianna*, significantly overrepresented codons reflect conserved codon preferences within the *Tulipa* genus. However, only one unique and overrepresented codon was specific to the *T. alberti* and *T. dubia* chloroplast genomes, indicating species-specific evolutionary dynamics. Furthermore, the analysis of underrepresented codons revealed species-specific trends. *T. greigii* and *T. kaufmanianna* exhibited a larger number of underrepresented codons compared to *T. alberti* and *T. dubia*, indicating potential differences in codon bias and evolutionary constraints between the species. Analysis of amino acid frequencies revealed that leucine was the most abundant amino acid in all *Tulipa* species, which is consistent with a previous study (Li et al., 2021c) on chloroplast genomes. Conversely, cysteine was the least abundant amino acid in *T. greigii*, *T. kaufmanianna*, and *T. dubia*, while methionine was the least frequent in *T. alberti*. Overall, the codon usage patterns observed in this study suggest a complex interplay between evolutionary forces, functional constraints, and environmental adaptation in shaping the chloroplast genomes of *Tulipa* species. Further research is needed to elucidate the precise mechanisms underlying these codon usage patterns and their implications for the biology and conservation of these endangered species.

### 4.4 Phylogenetic analysis

The evolutionary development of *Tulipa* species has been extensively studied through phylogenetic analysis. Phylogenetic studies use various methods, including molecular data analysis and morphological comparisons, to understand the evolutionary relationships among species within the genus. These studies aim to elucidate the genetic ancestry, divergence patterns, and evolutionary history of *Tulipa* species, shedding light on their evolutionary trajectories and the factors driving their diversification.

The results of the phylogenetic analysis revealed interesting patterns in the relationships among the 40 sequences from 25 species. The analysis indicates that the sequences were grouped into four major clusters, suggesting a significant degree of genetic variation among the species. These clusters likely represent evolutionary connections and may signify different evolutionary lineages or adaptations. Groups 1, 3, and 4 all contain species from the subgenus *Tulipa*. This indicates that the species within this subgenus are relatively closely related compared to species from other subgenera. This clustering is consistent with the idea that subgenus *Tulipa* represents a coherent evolutionary unit. Group 2, which contains species from subgenus *Orithyia* and *Eriostemons*, is separate from the groups containing *Tulipa* species. This separation highlights distinct evolutionary pathways or other three adaptations that have occurred in these subgenera.

The separation of *T. greigii* from other three *Tulipa* species and its clustering with species from subgenus *Orithyia* and *Eriostemons* is particularly noteworthy. It suggests that *T. greigii* might have a unique evolutionary history or genetic background that sets it apart from other *Tulipa* species, even though it belongs to the same genus. This could be due to historical geographic isolation, specific ecological adaptations, or other evolutionary pressures. These findings collectively contribute to our understanding of *Tulipa* species evolutionary relationships and highlight the impact of geographical and ecological factors on their genetic diversity and divergence. Further studies incorporating larger datasets and refined methodologies will continue to enhance our knowledge of *Tulipa* evolution and its underlying mechanisms.

## 5 Conclusion

In conclusion, this study achieved its objectives by determining and characterizing the complete chloroplast genome sequences of *T. greigii*, *T. kaufmanniana*, *T. alberti*, and *T. dubia* from Kazakhstan. Through this, we gained insights into the overall evolutionary dynamics of *Tulipa* chloroplast genomes. Our research also aimed to provide novel chloroplast markers for species identification within the genus *Tulipa*. We identified ten genes with effective polymorphic SSRs and high nucleotide diversity, serving as valuable molecular markers for *Tulipa* species identification. Furthermore, by comparing our sequences with published data from the NCBI database, we calibrated the phylogenetic position of *T. alberti*, *T. kaufmanniana*, *T. greigii*, and *T. dubia*, contributing to a more comprehensive understanding of *Tulipa* phylogeny. The newly identified chloroplast genes with high SSRs offer valuable tools for species identification and population studies, aiding conservation efforts and future *Tulipa* genetics and evolution research.

## Data Availability

The datasets generated and analyzed for this study can be found in the NCBI database (https://www.ncbi.nlm.nih.gov/) under the accession numbers OR458821, PP329299, PP335814, and OR662047.
