# Supplementary material for: Kazakhstan tulips: comparative analysis of complete chloroplast genomes of four local and endangered species of the genus Tulipa L
Source: Front Plant Sci. 2024 Nov 12;15:1433253. doi: 10.3389/fpls.2024.1433253 (PMC11588485; doi:10.3389/fpls.2024.1433253)
Supplement: Supplementary file 1 [file DataSheet1.pdf]

Figure S1: The results of the number of identical base pair frequencies, base pairs undergoing transitions, and base pairs undergoing transversion in all four genomes of four *Tulipa* species

*Directional Pair Frequencies

```
Data Filename: 4-21.10.2024 Whole genome alighnment last version.meg
Data Title: 4
Nucleotide Pair Frequencies
Sites Used: All selected
No. of sequences used: 4
Sequences used
  NC 084336.1 Tulipa dubia
  NC 087048.1 Tulipa greigii chloroplast complete genome
  NC 087786.1 Tulipa kaufmanniana
  OR458821 T alberti Chloroplast complete genome
All frequencies are averages (rounded) over all taxa.
ii = Identical Pairs
si = Transitionsal Pairs
sv = Transversional Pairs
R  = si/sv
Domain        ii     si     sv     R      TT     TC     TA     TG     CT     CC     CA     CG     AT     AC     AA     AG     GT     GC     GA     GG Total  Domain Info
   1. Avg 150926    288    264    1,1  48388     77     46     39     73  28166     43      5     50     39  47200     72     38      5     66  27172 151477,2  Data
    . 1st  50305     99     81    1,2  15874     28     15     10     27   9486     13      1     15     12  15598     22     12      2     21   9346  50484,7  1st Pos Data
    . 2nd  50319     88     84    1,1  15976     22     16     14     20   9434     12      1     16     11  16034     25     13      1     21   8874  50490,5  2nd Pos Data
    . 3rd  50302    101    100    1,0  16537     27     14     14     26   9246     18      3     19     16  15568     24     13      2     24   8952  50502,0  3rd Pos Data
```

Table S1: Lengths of introns in whole chloroplast genomes of four *Tulipa* species

	T. alberti	*T. kaufmanniana*	*T. greigii*	*T. dubia*
Introns	Intron length (bp)			
ndhA	1011	1011	**1012**	1011
trnA-UGC-2	795	795	795	795
trnI-GAU-2	930	930	930	930
rps12B	859	**864**	847	**864**
rps12B	540	540	540	540
ndhB	692	692	692	692
rpl2	651	651	**662**	651
rpl16	744	744	**750**	744
petD	779	779	779	779
petB	806	806	805	806
clpP1	**817**	**817**	808	602
clpP1	597	596	604	**817**

rps12A	587	587	**589**	587
trnV-UAC	590	590	590	590
trnL-UAA	**572**	**572**	568	568
pafI	721	721	**733**	713
pafI	713	713	713	721
rpoC1	691	692	685	**719**
atpF	797	798	**800**	**800**
trnG-UCC	721	721	724	**727**
rps16.	**879**	**879**	874	874
trnK-UUU	2605	2605	2606	**2620**
rpl2-2	651	651	**662**	651
ndhB-2	692	692	692	692
rps12B-2	859	**864**	847	**864**
rps12B-2	540	540	540	540
trnI-GAU	930	930	930	930
trnA-UGC	795	795	795	795

Length introns are in bold.

Table S2: Frequency of Identified SSRs in whole chloroplast genomes of four *Tulipa* species

**Taxonomy**	**Total**	**SSC**	**IR**	**LSC**	**Mono**	**Di**	**Tri**	**Tetra**	**Penta**	**Hexa**
T. alberti	158	31	26	102	56	38	53	8	1	2
T. kaufmanniana	157	30	28	100	57	37	53	8	0	2
T. greigii	173	32	26	116	70	39	51	10	3	0
T. dubia	163	30	28	100	61	38	53	7	0	3

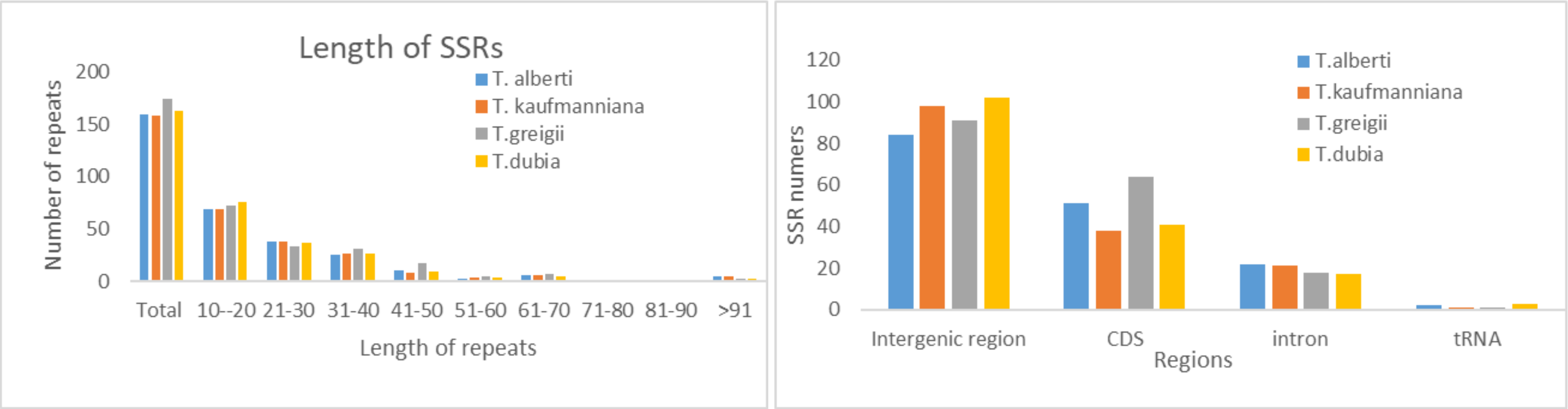

Figure S2: SSR repeat length and distribution in different regions of the genomes of four *Tulipa* species.

Table S3: 10 genes which has effective polymorphic SSRs with high sequence variability (SV>2,5814)

**No.**	**Region**		**Genes**	**Length (bp)**	**Sequence variability (SV)**
1	SSC	CDS	*ndhF*	305	4,1115
2	LSC	CDS	*ycf1*	5313	5,8140
3	LSC	CDS	*rpl22*	410	3,8835
4	LSC	CDS	*rps3*	656	3,6585
5	LSC	CDS	*rpl36*	113	3,5088
6	LSC	CDS	*psbH*	221	5,3812
7	LSC	CDS	*cemA*	690	3,0435
8	LSC	CDS	*rbcL*	1442	3,3626
9	LSC	CDS	*rpoC2*	4143	2,5814
10	LSC	CDS	*matK*	1538	6,1024

Table S4: Codon content of 20 amino acids and stop codons in CDS of the genomes of four *Tulipa* species

			T. albertii		***T. greigii***		***T. kaufmanianna***		***T. dubia***	
			Number	RSCU	Number	RSCU	Number	RSCU	Number	RSCU
1	Alanine	GCT	176	1,15	621	1,8	612	1,8	138	0,98
		GCC	113	0,74	215	0,62	212	0,62	111	0,79
		GCA	213	1,39	404	1,17	408	1,2	188	1,34
		GCG	111	0,72	138	0,4	129	0,38	125	0,89
2	Arginine	CGT	135	0,47	353	1,39	339	1,4	100	0,49
		CGC	117	0,4	95	0,37	89	0,37	77	0,38
		CGA	242	0,84	342	1,34	326	1,34	192	0,94
		CGG	227	0,78	110	0,43	106	0,44	219	1,07
		AGA	632	2,18	493	1,94	466	1,92	393	1,92
		AGG	385	1,33	134	0,53	130	0,54	247	1,21
3	Asparagine	AAT	792	1,33	1005	1,57	890	1,56	595	1,25
		AAC	402	0,67	273	0,43	249	0,44	360	0,75
4	Aspartic acid	GAT	420	1,35	843	1,62	792	1,62	372	1,33
		GAC	202	0,65	199	0,38	185	0,38	187	0,67
5	Phenylalanine	TTT	1067	1,23	948	1,3	862	1,26	814	1,1
		TTC	675	0,77	512	0,7	501	0,74	663	0,9
6	Leucine	TTA	610	1,45	889	2	832	1,98	625	1,13
		TTG	551	1,31	515	1,16	493	1,17	760	1,38
		CTT	520	1,24	584	1,31	561	1,33	573	1,04
		CTC	249	0,59	171	0,38	159	0,38	391	0,71
		CTA	342	0,81	357	0,8	329	0,78	536	0,97
		CTG	247	0,59	157	0,35	148	0,35	429	0,78
7	Isoleucine	ATT	850	1,2	1086	1,44	1025	1,46	744	1,02
		ATC	584	0,82	423	0,56	407	0,58	602	0,82
		ATA	694	0,98	747	0,99	681	0,97	849	1,16
8	Methiodine	ATG	485	1	606	1	570	1	752	1

9	Valine	GTT	408	1,45	518	1,48	497	1,47	454	1,24
		GTC	222	0,79	185	0,53	177	0,52	266	0,73
		GTA	304	1,08	514	1,46	495	1,47	437	1,2
		GTG	191	0,68	187	0,53	180	0,53	305	0,83
10	Serine	TCT	502	1,22	574	1,71	549	1,73	392	1,47
		TCC	357	0,87	298	0,89	287	0,9	239	0,9
		TCA	612	1,19	433	1,29	402	1,27	402	1,51
		TCG	334	0,81	181	0,54	172	0,54	272	1,02
		AGT	349	0,85	418	1,25	401	1,26	191	0,72
		AGC	307	0,75	106	0,32	93	0,29	103	0,39
11	Proline	CCT	213	1,01	412	1,54	396	1,55	212	0,91
		CCC	163	0,77	222	0,83	213	0,84	189	0,81
		CCA	296	1,4	318	1,19	293	1,15	299	1,28
		CCG	175	0,83	119	0,44	117	0,46	231	0,99
12	Threonine	ACT	264	0,95	532	1,63	508	1,65	211	1,03
		ACC	246	0,88	220	0,68	205	0,67	174	0,85
		ACA	401	1,44	413	1,27	385	1,25	291	1,42
		ACG	206	0,74	137	0,42	133	0,43	145	0,71
13	Tyrosine	TAT	828	1,32	793	1,63	739	1,63	600	1,24
		TAC	422	0,68	182	0,37	165	0,37	368	0,76
14	Histidine	CAT	360	1,31	494	1,6	471	1,6	316	1,22
		CAC	191	0,69	124	0,4	118	0,4	202	0,78
15	Glutamine	CAA	514	1,35	683	1,52	629	1,49	508	1,17
		CAG	247	0,65	216	0,48	214	0,51	359	0,83
16	Lysine	AAA	988	1,31	1034	1,49	864	1,46	834	1,16
		AAG	525	0,69	350	0,51	322	0,54	605	0,84
17	Glutamic acid	GAA	553	1,31	1010	1,51	917	1,49	557	1,23
		GAG	290	0,69	331	0,49	315	0,51	346	0,77
18	Cysteine	TGT	394	1	216	1,48	207	1,48	239	1,15
		TGC	391	1	76	0,52	73	0,52	175	0,85
19	Tryptophen	TGG	559	1	447	1	418	1	424	1

20	Glycine	GGT	226	0,73	550	1,27	547	1,29	245	0,92
		GGC	227	0,73	196	0,45	185	0,44	160	0,6
		GGA	434	1,4	691	1,6	668	1,57	343	1,28
		GGG	355	1,14	295	0,68	298	0,7	321	1,2
			24095		25695		24154		22457	

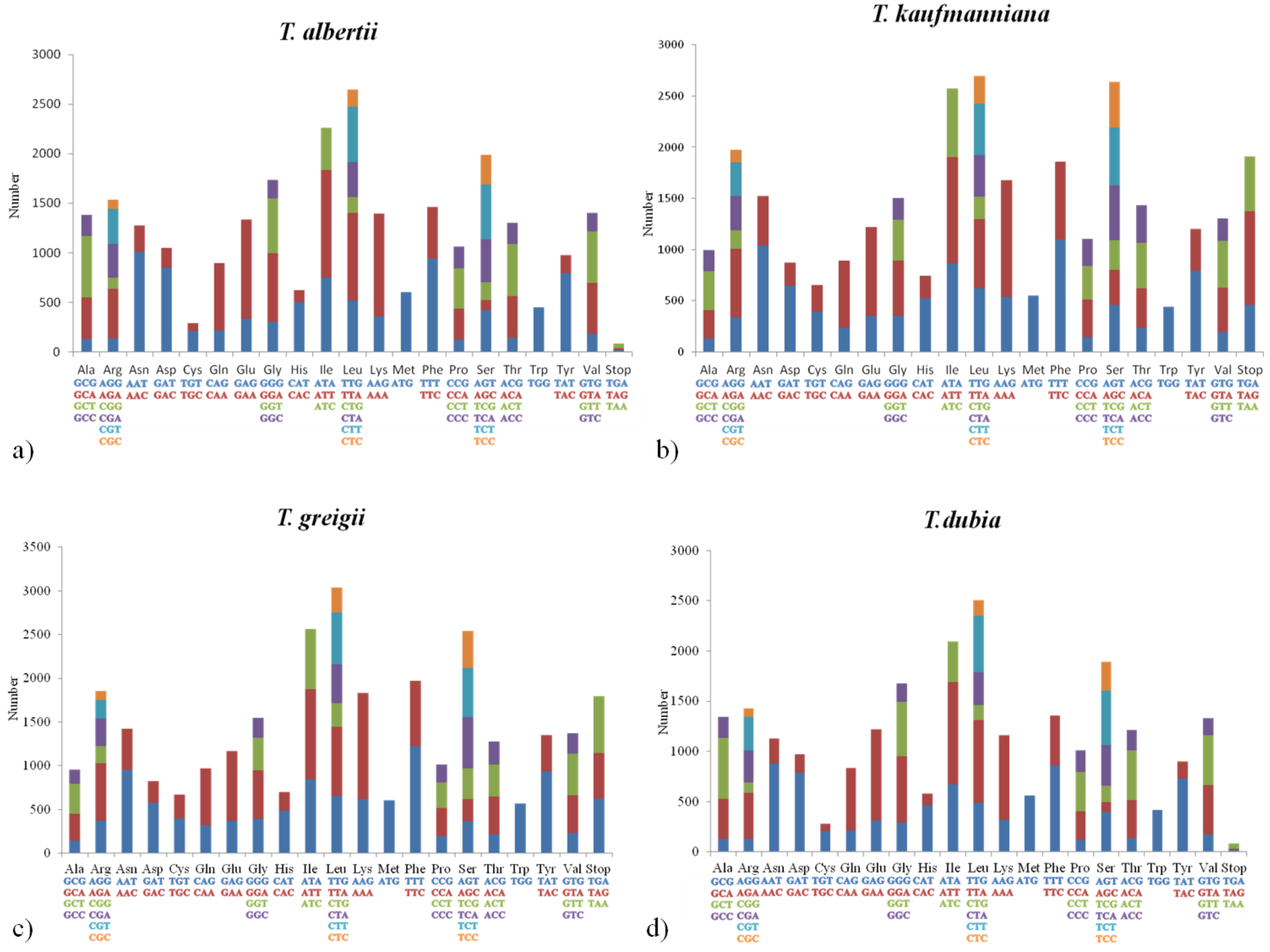

Figure S3: Codon content of 20 amino acids and stop codons in CDS of the genomes of four *Tulipa* species
